# Clinical associations of temporomandibular disorder and bruxism related symptoms with periodontal disease progression

**DOI:** 10.3389/froh.2025.1620861

**Published:** 2025-10-21

**Authors:** Nils Werner, Katrin Heck, Christina Ern, Richard Heym, Vinzenz Le, Oliver Schubert, Charlotte Wetzel, Vinay Pitchika, Falk Schwendicke, Matthias Folwaczny, Caspar Victor Bumm

**Affiliations:** 1 Department of Conservative Dentistry, Periodontology and Digital Dentistry, LMU University Hospital, LMU Munich, Munich, Germany; 2 Private Practice, Munich, Germany; 3 Department of Prosthetic Dentistry, University Hospital, LMU Munich, Munich, Germany

**Keywords:** periodontal disease progression, tooth loss, temporomandibular disorders, periodontitis, bruxism

## Abstract

**Introduction:**

The aim of this study was to analyse whether symptoms of temporomandibular disorders (TMD) or bruxism were associated with the progression of periodontitis. A potential association could be explained by a decreased level of oral hygiene in patients presenting with orofacial pain.

**Materials and methods:**

148 patients diagnosed with periodontitis received individual department specific screening for symptoms of TMD or bruxism prior to initial treatment and were stratified into patients with symptoms related to TMD or bruxism (STMDoB = 30) and without symptoms (NO_STMDoB = 118). Progression of periodontitis was determined by tooth loss (TL) as well as radiographic bone loss (RBL), using longitudinal radiographic data with a follow-up of at least 5 years.

**Results:**

Patients presented with a median of 60 [52;68] years, 25 [21;27] teeth and a mean RBL of 50.5 ± 16.4% not showing difference among both study groups. Neither RBL [1.2 [0.0;6.0] % STMDoB vs. 2.9 [0.0;9.1] % NO_STMDoB, *p* = 0.165] nor TL [1 [0;3] STMDoB vs. 1 [0;3] NO_STMDoB; *p* = 0.195] differed significantly between both study groups, with equally low periodontal progression in both groups. Regression models revealed no association of any reported symptom of TMD or bruxism with periodontal progression (*β*: 9.07; CI: −4.09;22.23; *p* = 0.446 for RBL and rate ratio: 1.09; CI: 0.80;1.47; *p* = 0.587 for TL).

**Conclusions:**

The present data showed no association of STMDoB with periodontal disease progression on the patient level.

## Introduction

Periodontitis is a chronic, multifactorial inflammatory disease associated with a dysbiotic biofilm that leads to destruction of the tissues surrounding the tooth and ultimately to tooth loss (1). Consequently, the primary goal of periodontal therapy is the long-term retention of diseased teeth (2). In this context, proper infection control is essential to prevent further destruction of the periodontal tissues (3).

The standard anti-inflammatory therapy is a cause-related non-surgical periodontal debridement of the root surface (3). Thorough treatment, which is continued over time as supportive periodontal care (SPC), prevents further attachment loss and tooth loss (4–6). However, it has been shown that the rate of progression of periodontitis varies greatly between individuals. Several factors have been identified that increase the risk of periodontal disease progression and tooth loss (4, 7). These include smoking (8–10), diabetes (11, 12) and irregular adherence with SPC (7).

On the other hand, temporomandibular disorders (TMD) are a group of conditions that describe dysfunctions in the temporomandibular joint. These conditions occur in 20%−40% of adults reaching the highest prevalence in 20–40-year-olds (13). Typical symptoms include facial myalgia and joint clicking (13, 14). TMD is frequently linked to bruxism, which can manifest in wear facets (15) and is often associated with non-physiological or even excessive occlusal forces (16). In this context, occlusal trauma might cause increased tooth mobility. This so-called jiggling trauma can induce resorption of the tooth supporting alveolar bone and may be found around teeth with or without periodontitis. In the latter case, the term “secondary occlusal trauma” has been established, suggesting a traumatic occlusion at teeth with a reduced attachment level. A possible association between periodontitis and TMD is subject of discussion since many years but yet has still not conclusively confirmed (17). A recent narrative review by Fan et al. concluded that malocclusion is not a trigger for plaque-induced disease and has uncertain impact on on periodontal disease progression (16). On the contrary, Sonnenschein et al. clearly showed that splinting teeth showing increased mobility improves the outcomes of non-surgical periodontal therapy (18). Furthermore, for stage IV periodontal disease, occlusal adjustment and splinting of the teeth are considered in case of a secondary occlusal trauma (19, 20).

Yet, the majority of studies on this issue have focused exclusively on the impact of occlusal trauma on the tooth level, neglecting to consider the association of the broader clinical picture of TMD and dysfunctional masticatory activity, i.e., bruxism on the progression of periodontitis considering the patient level.

Accordingly, the objective of this study was to comprehensively evaluate the association of TMD or bruxism related symptoms on the periodontal status at baseline (1) and on the progression of the disease following the first two steps of periodontal therapy after five years (2).

## Methods

Data were collected as part of a prospective clinical study and analyzed retrospectively. The studies were reviewed and approved by the Ethics Committee of the Medical Faculty of the Ludwig-Maximilians-University, Munich [No. 025-11 (prospective cohort), No. 22-0669 (retrospective analysis)]. The cohort study was conducted in accordance with good clinical practice and the Declaration of Helsinki, as revised in 2013, and was registered in the German Clinical Trials Register (DRKS00028923). The study description follows the guidelines for reporting observational studies (STROBE) (21).

### Study population

The original study cohort comprised 759 patients receiving steps I and II of periodontal therapy for the first time or for retreatment due to recurrent disease. All patients were treated between 2011 and 2016 in the undergraduate course at the Department of Conservative Dentistry and Periodontology, University Hospital, LMU Munich (22).

For enrollment into the study the following criteria were used: (1) Age ≥18 years, (2) diagnosis of periodontitis or recurrent disease according to the latest classification (23) (3) periodontal chart documenting probing pocket depth (PPD) and bleeding on probing (BOP) at six sites/tooth before steps I and II of periodontal therapy, (4) panoramic radiographs at baseline and at least 5 years after re-evaluation, (5) screening for TMD and bruxism related symptoms (STMDoB) at baseline, and (6) capacity and willingness to provide written informed consent. The exclusion criteria were: (1) pregnancy at baseline, (2) previous periodontal treatment <2 years before enrollment into the study, and (3) ongoing SPC at baseline.

### Clinical parameters and outcome variables

As described in detail before (22), all examinations were conducted prior to the first two steps of periodontal therapy (T0) by two calibrated periodontists (CE and RH) (24). PPD were measured using the PCP-12 probe in whole mm at 6 points per tooth and checked for BOP after 30 s (25). Tooth mobility was documented using the classification of mobility as described by Miller et al. (26), and transferred to a patient-related variable by indicating the proportion of teeth with mobility >class 1 out of the total dentition as well as their number. Periodontal pockets were defined as sites with PPD >3 mm, and deep periodontal pockets as PPD ≥6 mm. Proportions of all periodontal pockets and deep periodontal pockets were included as variables on the patient level, calculated as % of sites with corresponding pockets. Regular SPC was assumed if the patient attended at least once a year. All patients received a standardized department specific screening for TMD or bruxism using an individual protocol focusing on physical signs and symptoms. In brief, the following parameters were examined: (1) muscle or facial pain upon opening or closing, (2) noise of the temporomandibular joint during function (i.e., creaking or clicking), (3) wear facets of the teeth, (4) traumatic occlusion, (5) limited mouth opening <40 mm, (6) deviation upon opening. If more than two of these symptoms were detected, the patient was classified as STMDoB herein (27). Treatment of TMD and bruxism included systemic medication such as NSARs, various types of splints as standard, and physiotherapy. Patients were radiographically re-evaluated once, at a mean follow-up of 72 months. No interim follow-up visits were conducted. No further assessment of STMDoB was performed during the observation period. As outcome parameter periodontal progression was determined by tooth loss (TL) as well as radiographic bone loss (RBL) were defined using longitudinal radiographic data over at least 5 years.

### Radiographic analysis

Radiographic analysis was described in detail before (28). For each patient, two panoramic radiographs were analyzed, taken exclusively as indicated, with a minimum time interval of 5 years. Evaluation of all radiographs was independently conducted by two raters (NW, CVB) following a calibration phase. This calibration phase entailed 20 panoramic radiographs that were not included into the study, with the objective of ensuring inter-rater reliability. The intra-rater reliability was subsequently assessed by re-evaluating 20 randomly selected radiographs from the study cohort. In case of discrepancy of >10% between the measurements of RBL, the radiographs were collectively re-examined until a consensus was reached. To circumvent any potential misinterpretation of RBL that might arise from variations in radiographic angles, teeth exhibiting a measured root length deviation exceeding 1 mm between the baseline and the follow-up were excluded from the study. The digital radiographs were subjected to analysis using dedicated imaging software (Sidexis XG 2.63, Dentsply Sirona, Bensheim, Germany). The landmarks used in this study were defined as previously described by Nibali et al., and included the cemento-enamel junction (CEJ), the radiographic apex, and the base of the alveolar bone crest (bone level, BL) (29). The distances between the cementoenamel junction (CEJ) and the apex (CEJ-apex) and between the CEJ and the buccal (CEJ-BL) were measured to the nearest mm at the most severely affected tooth. The percentage root-buccal length (RBL) was calculated by dividing CEJ-BL/CEJ-apex (29, 30). In instances where the CEJ was not available due to the presence of prosthodontic restorations, the restoration margin served as an alternative landmark to the CEJ.

### Sample size

Due to the retrospective nature of this study, an *a priori* sample size calculation was not feasible; instead, the available dataset determined the sample size. Consequently, a *post hoc* power analysis was performed using G*Power (version 3.1), based on the R² value obtained from the final multiple linear regression model (R² = 0.266). Additional parameters included the number of predictors, a significance level of 0.05, and the actual sample size. This analysis yielded a calculated power of 99%. However, to avoid overestimating the power due to the R² value from a fully optimised model, we additionally calculated the power specifically for our primary predictor of interest (STMDoB) using its partial R² value (0.0064). Furthermore, we conducted an additional power analysis based on the R² value (0.003) obtained from a simple linear regression model that included only the primary predictor.

### Source of bias

The retrospective design of this study increases the risk of bias, including issues related to measurements, interventions, and data interpretation. To address potential classification and performance biases within the undergraduate program, supervisory operators (CE and RH) were pre-calibrated in stages, achieving a *κ*-value of 0.82 (24). Likewise, radiographic examination was performed separately by two calibrated examiners (CB and NW, r = 0.96) to mitigate misclassification bias (28).

### Statistical analysis

The normality of data was tested using the Kolmogorov-Smirnow test. Data that were normally distributed were given as mean ± standard deviation (SD), whilst non-normally distributed data were given as median and interquartile range [q1;q3]. Categorical data were presented as absolute numbers and relative frequencies (percentages). Patients were stratified according to the presence of TMD symptoms at baseline. Differences between subject groups were compared using Student's *t*-test for normally distributed variables, Mann–Whitney *U*-test for ordinal or skewed variables, and chi-squared or Fisher Exact test for categorical variables. Linear regression models were used to detect an association between STMDoB and RBL, and Poisson regression models for STMDoB and TL. To adjust for potential confounders, these were included in both models. Results are presented as adjusted β-coefficients per percentage RBL or adjusted rate ratios per TL with corresponding 95% confidence intervals (CIs). The significance level was set at α = 0.05 for all tests. To adjust for multiple testing, the Bonferroni procedure was used for subgroup analysis and the significance level was corrected to α = 0.025. Data were routinely checked for plausibility; no implausible outliers were identified. All analyses were performed with SPSS (version 29.0, IBM, Armonk, USA).

## Results

### Patient characteristics

**Figure 1:**
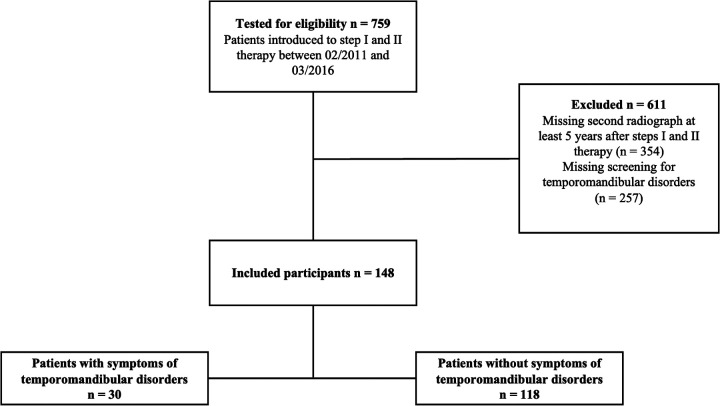
Flow diagram of the process of trial inclusion/exclusion.

Seven hundred fifty-nine patients received steps I and II of periodontal therapy between February 2011 and March 2016. The final analysis included 148 patients who met the inclusion criteria described above (Figure 1). This study cohort presented with a median number of 25 [21;27] teeth at a median age of 60 [52;68] years at baseline, the male-to-female ratio was 54.7/45.3%, 19.6% of the patients were current smokers at baseline, and 8.9% were diagnosed with diabetes mellitus (Table 1). Of the total of 148 subjects evaluated; 1 (0.7%) exhibited stage I, 20 (13.5%) stage II, 78 (52.7%) stage III and 49 (33.1%) stage IV periodontitis. Concerning the periodontal grade, 2 participants (1.4%) were classified as grade A, 91 (61.5%) as grade B, and 55 (37.2%) as grade C. 118 participants showed NO_STMDoB, while 30 reported on STMDoB (Table 1). In the STMDoB group, 11 participants (36.7%) reported signs of myalgia, 12 (40%) noises from the temporomandibular joint, 26 (86.7%) wear facets, 17 (56.6%) traumatic occlusion, 2 (6.7%) limitations on mouth opening, and 7 (23.3) a deviation to the ipsilateral side upon opening (Table 1). Of the patients in the STMDoB group, 6 (20.7%) had a bite splint and 10 (34.5%) received physiotherapy specially adapted to TMD (Table 1).

**Table 1:** Patient characteristics.

Variable	All	STMDoB	NO_STMDoB	*p*-value
*N*	148	30	118	
Age, years	60 [52;68]	62 [52;67]	60 [52;69]	0.958
Male sex, *n* (%)	67 (45.3)	12 (40.0)	55 (46.6)	0.516
Smoker, *n* (%)	29 (19.6)	8 (26.7)	21 (17.8)	0.166
Diabetes mellitus, *n* (%)	13 (8.9)	4 (13.3)	9 (7.6)	0.517
SPT compliance, *n* (%)	52 (42.6)	10 (41.7)	42 (42.9)	0.916
Teeth, *n* [q1;q3]	25 [21;27]	26 [20;28]	26 [20;28]	0.886
Stage, *n* (%)				
I	1 (0.7)	1 (3.3)	0 (0)	0.256
II	20 (13.5)	4 (13.3)	16 (13.6)	
III	78 (52.7)	16 (53.3)	62 (52.5)	
IV	49 (33.1)	9 (30.0)	40 (33.9)	
Grade, *n* (%)				
A	2 (1.4)	1 (3.3)	1 (0.8)	0.562
B	91 (61.5)	20 (66.7)	83 (70.3)	
C	55 (37.2)	9 (30.0)	34 (28.8)	
Muscle and TMJ pain, *n* (%)	12 (8.1)	11 (36.7)	1 (0.8)	**<0** **.** **001**
TMJ noises during function, *n* (%)	17 (11.5)	12 (40.0)	5 (4.2)	**<0**.**001**
Wear facets, *n* (%)	76 (51.4)	26 (86.7)	50 (42.4)	**<0**.**001**
Traumatic occlusion, *n* (%)	17 (11.5)	17 (56.6)	0 (0)	**<0**.**001**
Assisted opening <40 mm, *n* (%)	3 (2.0)	2 (6.7)	1 (0.8)	**0**.**043**
Deviation to the ipsilateral side on opening, *n* (%)	11 (7.4)	7 (23.3)	4 (3.4)	**<0**.**001**
Splint, *n* (%)	15 (10.1)	6 (20.7)	9 (8.9)	0.083
Physical therapy, *n* (%)	26 (17.6)	10 (34.5)	16 (15.8)	0.079

Data are presented as median [q1; q3] or frequency (%). *p*-values are calculated using Chi-square-test, Fisher exact test or Mann–Whitney *U*-test. NO_STMDoB, no symptoms of temporomandibular disorders or bruxism; SPT, supportive periodontal therapy; STMDoB, symptoms of temporomandibular disorders or bruxism. Bold indicates statistically significant values (*p* < 0.05).

In terms of periodontal characteristics, both groups were relatively similar. Only a slightly but significantly higher PI was found in the group of STMDoB patients [STMDoB: 54.0 [42.9;66.8] vs. NO_STMDoB: 45.4 [19.1;62.4], *p* = 0.027] (Table 2). Within the group with STMDoB, no significant difference was found at baseline between patients with and without treatment for TMD or bruxism in terms of periodontal parameters (Table 2.1).

**Table 2:** Clinical parameters of periodontitis.

Variable	All	STMDoB	NO_STMDoB	*p*-value
*N*	148	30	118	
PPD%, %	14.8 [7.3;27.5]	14.2 [7.5;29.9]	14.9 [7.0;27.3]	0.987
PPD_Deep%, %	2.1 [0.0;7.0]	1.6 [0.0;8.4]	2.5 [0.0;7.0]	0.729
BOP (all), %	31.1 [19.9;46.1]	31.4 [18.8;49.0]	25.5 [8.3;42.7]	0.073
PI (all), %	52.2 [40.6;65.1]	54.0 [42.9;66.8]	45.4 [19.1;62.4]	**0** **.** **027**
Teeth with mobility >I, *n*	1 [0;5]	3 [0;7]	1 [0;4]	0.085
Teeth with mobility >I, %	4.5 [0.0;21.7]	10.4 [0.0;33.1]	3.6 [0.0;17.7]	0.086
Boneloss at baseline, %	51.2 [37.3;62.3]	50.5 [38.5;58.2]	51.2 [37.1;17.7]	0.989
Toothloss during OP	1 [0;3]	2 [0;3]	1 [0;3]	0.220
Toothloss per year during OP	0.14 [0.00;0.43]	0.23 [0.00;0.51]	0.11 [0.00;0.43]	0.158
Boneloss during OP, %	2.7 [0.0;8.4]	1.2 [0.0;6.0]	2.9 [0.0;9.1]	0.165
Boneloss after OP,%	57.7 [41.4;69.0]	53.2 [42.3;65.7]	58.8 [39.9;69.3]	0.650
No toothloss, *n* (%)	62 (41.9)	10 (33.3)	52 (44.1)	0.287
No boneloss during OP, *n* (%)	54 (36.5)	14 (46.7)	40 (33.9)	0.195

Data are presented as median [q1; q3] or frequency (%) unless stated otherwise. *p*-values are calculated using Chi-square-test, *t*-test, Fisher exact test or Mann–Whitney *U*-test BOP, bleeding on probing; NO_STMDoB, no symptoms of temporomandibular disorders or bruxism; PI, plaque index; PPD%, percentage of sites with probing depth >3 mm; PPD_Deep%, percentage of sites with probing depths >5 mm; OP, observation period; STMDoB, symptoms of temporomandibular disorders or bruxism. Bold indicates statistically significant values (*p* < 0.05).

**Table 2.1:** Clinical parameters of periodontitis.

Variable	All	STMDoB therapy	No STMDoB therapy	*p*-value
N	130	11	19	
PPD%, %	14.2 [7.5;29.9]	24.3 [13.5;33.3]	10.0 [5.1;16.7]	0.037
PPD_deep%, %	1.6 [0.0;8.4]	5.8 [0.6;11.1]	1.0 [0.0;4.2]	0.103
BOP, %	31.4 [18.8;49.0]	30.8 [16.1;39.2]	36.1 [20.7;60.8]	0.471
PI, %	54.0 [42.9;66.8]	46.7 [28.7;51.1]	63.0 [48.8;67.4]	0.085
Teeth with mobility >I, *n*	3 [0;7]	2 [0;6]	3 [0;8]	0.899
Teeth with mobility >I, %	10.4 [0.0;33.1]	10.0 [0.0;22.2]	12.5 [0.0;37.5]	0.672
Boneloss at baseline, %	50.5 [38.5;58.2]	51.8 [43.5;75.6]	49.3 [35.7;57.2]	0.445
Toothloss during OP	2 [0;3]	0 [1;3]	2 [0;4]	0.216
Toothloss per year during OP	0.23 [0.00;0.51]	0.11 [0.00;0.50]	0.40 [0.00;0.71]	0.216
Radiographic boneloss during OP, %	1.2 [0.0;6.0]	−3.0 [−5.4;4.2]	2.8 [−0.7;14.7]	0.064
Radiographic boneloss after OP, %	53.2 [42.3;65.7]	50.0 [40.5;75.4]	56.1 [50.4;64.8]	0.611
No toothloss, *n* (%)	10 (33.3)	5 (45.6)	5 (26.3)	0.284
No boneloss during OP, *n* (%)	14 (46.7)	8 (72.7)	6 (31.6	0.029

Data are presented as median [q1; q3] or frequency (%) unless stated otherwise. *p*-values are calculated using Chi-Square-test, *t*-test or Mann–Whitney *U*-test. Bonferroni correction applied for multiple testing; significance level adjusted to α = 0.025. BOP, bleeding on probing; PI, plaque index; PPD%, proportion of sites with probing depth >3 mm; PPD_Deep%, propotion of sites with probing depths >5 mm; OP, observation period; STMDoB, symptoms of temporomandibular disorders or bruxism.

### Periodontal disease progression

The mean observation period (i.e., the time between panoramic radiographs) was 72 (± 12) months. During the observation period, 10 patients (41.7%) of the STMDoB group and 42 patients (42.9%) of the NO_STMDoB group attended regular SPC appointments (Table 1). Periodontal progression as measured by RBL was not significantly different between the two stratified groups [STMDoB: 1.2 [0.0;6.0] % vs. NO_STMDoB: 2.9 [0.0;9.1] %, *p* = 0.165]. TL was equally low in both groups and showed no significant difference in TL per patient [STMDoB: 2 [0;3] vs. NO_STMDoB: 1 [0;3], *p* = 0.220] or TL per patient per year [STMDoB: 0.23 [0.00;0.51] vs. NO_STMDoB: 0.11 [0.00;0.43], *p* = 0.158] (Table 2). A subgroup analysis of patients with STMDoB showed that patients treated for TMD had a reduced RBL, although this was not statistically significant (Table 2.1).

Regression analysis provided further support for these findings. A linear regression model adjusted for potential confounders (sex, age, diabetes, baseline RBL and baseline smoking status) was used to model a potential association between STMDoB and RBL. It showed no association between periodontal progression and STMDoB [*β*: 9.07; CI:−4.09;22.23; *p* = 0.446 (Table 3)]. In addition, no association between TL and STMDoB was found in a Poisson regression model adjusted for the same confounders (rate ratio: 1.09; CI:0.80;1.47; *p* = 0.587 Table 4).

**Table 3:** Linear regression model—dependent variable radiographic boneloss during OP.

Variable (reference categorie)	Multivariate linear regression	
	%RBL (95% CI)	*p*-value
Sex (female)	−1.47 (−12.67;9.73)	0.788
Smoking status (smoker)	2.27 (−8.81;9.73)	0.675
Diabetes (diabetic)	−10.51 (−38.58;17.56)	0.446
Baseline radiographic boneloss	−0.38 (−0.76;−0.00)	**0** **.** **049**
STMDoB (with STMDoB)	9.07 (−4.09;22.23)	0.446

Data are presented as β-Coefficient with corresponding 95% confidence interval (CI). Model calculated for all 148 patients. %RBL, radiographic boneloss during observation period; OP, observation period; STMDoB, symptoms of temporomandibular disorders or bruxism. Bold indicates statistically significant values (*p* < 0.05).

**Table 4:** Poisson regression model—dependent variable toothloss during OP.

Variable (reference categorie)	Multivariate Poisson regression	
	Toothloss (95% CI)	*p*-Value
Sex (female)	0.64 (0.50;0.82)	**<0** **.** **001**
Smoking status (smoker)	1.63 (1.20;2.21)	**0**.**002**
Diabetes (diabetic)	0.91 (0.55;1.53)	0.912
Baseline radiographic boneloss	0.98 (0.98;0.99)	**<0**.**001**
STMDoB (with STMDoB)	1.09 (0.80;1.47)	0.587

Data are presented as rate ratio with corresponding 95% confidence interval (CI). Model calculated for all 148 patients. OP, observation period; STMDoB, symptoms of temporomandibular disorders or bruxism Bold indicates statistically significant values (*P* < 0.05).

## Discussion

The results of this study revealed no association between STMDoB and the progression of periodontitis, neither using RBL nor TL, as indicators for periodontal progression. Nevertheless, the study showed a slightly but significantly poorer oral hygiene in patients with STMDoB, which could also have potential influences on the onset of periodontitis and caries.

The primary objective of periodontal therapy is to ensure the long-term retention of natural teeth. Tooth loss, however, remains relatively uncommon among patients undergoing standardized periodontal treatment. Long-term studies report low rates of tooth loss in periodontitis patients (6, 11). In consideration of the comparatively short observation period of our study, we used a surrogate endpoint, namely the progression of the disease, which was measured by assessing the radiographically most affected tooth, as is typically done in periodontal grading. While, periapical radiographs are widely considered to represent the gold standard for the diagnosis of periodontal bone loss. In clinical practice panoramic radiographs are mostly employed for initial diagnosis and monitoring of periodontitis (31). To improve comparability between both radiographs linear distortion of the root length >1 mm led to exclusion from further analysis.

Assignment of subjects to the two study groups was performed using an individual protocol focusing on physical signs and symptoms of TMD and bruxism, closely related to typical TMD pain screener (27, 32–34). Using the presence of more than two key indicators as a threshold for classification offers a practical and comprehensive approach focused on observable symptoms indicating functional impairment. While effective for initial screening, this threshold may encompass a wide range of patients with varying TMD and bruxism severity. Consequently, it may not accurately reflect the individual severity of the disease. According to current guidelines only complete DC/TMD or advanced imaging allow in depth classification. The method we used nevertheless provides an efficient yet balanced approach for preliminary screening in a clinical setting.

Previous studies found some evidence to suggest that TMD could influence the development and progression of periodontitis (16). TMD is a group of symptoms associated with a dysfunction in the temporomandibular joint, that may affect various functions in the oral cavity (27). Bruxism is associated with the persistent exposure of one or more teeth to excessive occlusal forces and thus manifests with different oral and tooth related symptoms (13). It is commonly accepted that supraphysiological occlusal loading of teeth might interfere with the course of periodontitis on the tooth level. Consequently it seems sensible to consider whether functional disorders, i.e., TMD/bruxism, might also, influence the progression of periodontitis on the patient level (35, 36). Considering the primary cause for initiation and perpetuation of periodontitis poor oral hygiene measures might represent a plausible connection between both of these diseases (37, 38). Humphrey et al. showed that TMD patients insufficient interdental hygiene in many cases (39). This may be attributable to an impaired mouth opening frequently observed in patients with orofacial pain (6, 40) and is well in line with the current data demonstrating poorer oral hygiene among STMDoB patients.

Furthermore, there might be an association between tooth mobility and STMDoB. This could be explained by persistent occlusal trauma and excessive occlusal force, which might be present for many hours in TMD and bruxism, and can even histologically compromise the periodontal ligament (16). These forces could lead to bone and root resorption on one side and periodontal ligament elongation on the other. Further progression could result in tooth mobility and is often associated with a widened periodontal space on radiographs (15, 16, 41). However, further progressed periodontitis could also lead to secondary occlusal trauma, tooth loosening, tooth migration, loss of posterior support, and loss of masticatory function, which may result in discomfort regarding the temporomandibular joint (20). This is corroborated by previous studies that have shown more severe symptoms of TMD in patients with tooth loosening and premature tooth loss indicating a two way interrelation between periodontitis and TMD (36). According to a recent systematic review in periodontally compromised patients occlusal adjustments but not tooth splinting had a positive effect on clinical attachment loss (19). Notably, in our study, we did not find a significant difference in tooth mobility between patients in both study groups.

This study has several of limitations that might reduce its generalisability, applicability and transferability. Due to the monocentric observational setting, overall generalisability is limited. Further, the current study may have suffered from incomplete data collection, especially regarding periodontal charts on follow-ups. All steps of therapy were performed in the undergraduate program under the supervision of experienced periodontists. While this setup presents a limitation regarding the comparability of the data, it offers a realistic picture of daily dental practice (22, 42). Participants were only re-evaluated once radiographically at a mean follow-up of 72 months. No interim assessments of STMDoB or related treatments were conducted. Therefore, changes in symptom status or treatment effects during the follow-up period could not be captured and evaluated. A further limitation of the study is that the patients were undergoing non-surgical periodontal therapy only. Consequently, it is unlikely that stage III and IV patients could achieve stable therapy endpoints; however, they may stabilise and demonstrate an acceptable outcome (43). Additionally, it should be noted that the diagnostic chart of the DC/TMD was not entirely employed, but rather, an individual protocol focusing on the typical TMD or bruxism related symptoms was utilized which limits the comparability. This is not an uncommon practice, as modifications of the DC/TMD are frequently used as a preliminary screening tool before initiating a potential therapeutic intervention (27, 32–34). Although the overall model showed strong explanatory power, the *post hoc* power for the primary predictor (STMDoB), based on its partial and univariable R², was low (approximately 16%). This indicates that the effect of STMDoB alone may be modest, and the study may have been underpowered to detect it. While STMDoB was not statistically significant in this context, the result should be interpreted with caution and warrants validation in larger or prospective studies. Furthermore, the diagnosis of sleep bruxism was not further differentiated leading to a limited interpretation of occlusal trauma overnight.

## Conclusions

Within the limitations of this study, there was not confirmed any association between STMDoB and periodontal disease progression herein. The current findings indicate that STMDoB have no direct effect on the progression of periodontal disease on the patient level. However, when considering the interrelation between bot disease entities on the tooth level clinicians should consider treating both of these conditions together as part of a comprehensive strategy.

## Data Availability

The data analyzed in this study is subject to the following licenses/restrictions: The data that support the findings of this study are available from the corresponding author upon reasonable request. Requests to access these datasets should be directed to Nils.werner@med.uni-muenchen.de.

## References

[1] Chapple ILC Mealey BL Van Dyke TE Bartold PM Dommisch H Eickholz P Periodontal health and gingival diseases and conditions on an intact and a reduced periodontium: consensus report of workgroup 1 of the 2017 world workshop on the classification of periodontal and peri-implant diseases and conditions. J Periodontol. (2018) 89(Suppl 1):S74–s84. 10.1002/jper.17-0719 29926944

[2] Hirschfeld L Wasserman B. A long-term survey of tooth loss in 600 treated periodontal patients. J Periodontol. (1978) 49(5):225–37. 10.1902/jop.1978.49.5.225 277674

[3] Suvan J Leira Y Moreno Sancho FM Graziani F Derks J Tomasi C. Subgingival instrumentation for treatment of periodontitis. A systematic review. J Clin Periodontol. (2020) 47(S22):155–75. 10.1111/jcpe.13245 31889320

[4] Gaunt F Devine M Pennington M Vernazza C Gwynnett E Steen N The cost-effectiveness of supportive periodontal care for patients with chronic periodontitis. J Clin Periodontol. (2008) 35(8 Suppl):67–82. 10.1111/j.1600-051X.2008.01261.x 18724842

[5] Manresa C Sanz-Miralles EC Twigg J Bravo M. Supportive periodontal therapy (spt) for maintaining the dentition in adults treated for periodontitis. Cochrane Database Syst Rev. (2018) 1(1):Cd009376. 10.1002/14651858.CD009376.pub2 29291254 PMC6491071

[6] Graetz C Plaumann A Schlattmann P Kahl M Springer C Sälzer S Long-term tooth retention in chronic periodontitis - results after 18 years of a conservative periodontal treatment regimen in a university setting. J Clin Periodontol. (2017) 44(2):169–77. 10.1111/jcpe.12680 28028838

[7] Eickholz P Kaltschmitt J Berbig J Reitmeir P Pretzl B. Tooth loss after active periodontal therapy. 1: patient-related factors for risk, prognosis, and quality of outcome. J Clin Periodontol. (2008) 35(2):165–74. 10.1111/j.1600-051X.2007.01184.x 18199150

[8] McGuire MK Nunn ME. Prognosis versus actual outcome. III. The effectiveness of clinical parameters in accurately predicting tooth survival. J Periodontol. (1996) 67(7):666–74. 10.1902/jop.1996.67.7.666 8832477

[9] Preshaw PM Heasman L Stacey F Steen N McCracken GI Heasman PA. The effect of quitting smoking on chronic periodontitis. J Clin Periodontol. (2005) 32(8):869–79. 10.1111/j.1600-051X.2005.00779.x 15998271

[10] Tonetti MS. Cigarette smoking and periodontal diseases: etiology and management of disease. Ann Periodontol. (1998) 3(1):88–101. 10.1902/annals.1998.3.1.88 9722693

[11] Faggion CM Jr. Petersilka G Lange DE Gerss J Flemmig TF. Prognostic model for tooth survival in patients treated for periodontitis. J Clin Periodontol. (2007) 34(3):226–31. 10.1111/j.1600-051X.2006.01045.x 17257157

[12] Preshaw PM Bissett SM. Periodontitis and diabetes. Br Dent J. (2019) 227(7):577–84. 10.1038/s41415-019-0794-5 31605062

[13] Ferneini EM. Temporomandibular joint disorders (tmd). J Oral Maxillofac Surg. (2021) 79(10):2171–2. 10.1016/j.joms.2021.07.008 34620421

[14] Gauer RL Semidey MJ. Diagnosis and treatment of temporomandibular disorders. Am Fam Physician. (2015) 91(6):378–86. 25822556

[15] Stillman PR. What is traumatic occlusion and how can it be diagnosed and corrected? J Am Dent Assoc. (1925) 12(11):1330–8. 10.14219/jada.archive.1925.0304

[16] Fan J Caton JG. Occlusal trauma and excessive occlusal forces: narrative review, case definitions, and diagnostic considerations. J Periodontol. (2018) 89(S1):S214–S22. 10.1002/JPER.16-0581 29926937

[17] Jepsen S Caton JG Albandar JM Bissada NF Bouchard P Cortellini P Periodontal manifestations of systemic diseases and developmental and acquired conditions: consensus report of workgroup 3 of the 2017 world workshop on the classification of periodontal and peri-implant diseases and conditions. J Clin Periodontol. (2018) 45(Suppl 20):S219–s29. 10.1111/jcpe.12951 29926500

[18] Sonnenschein SK Ciardo A Kilian S Ziegler P Ruetters M Spindler M The impact of splinting timepoint of mobile mandibular incisors on the outcome of periodontal treatment-preliminary observations from a randomized clinical trial. Clin Oral Investig. (2022) 26(1):921–30. 10.1007/s00784-021-04075-4 PMC8311063 34309736

[19] Dommisch H Walter C Difloe-Geisert JC Gintaute A Jepsen S Zitzmann NU. Efficacy of tooth splinting and occlusal adjustment in patients with periodontitis exhibiting masticatory dysfunction: a systematic review. J Clin Periodontol. (2022) 49(Suppl 24):149–66. 10.1111/jcpe.13563 34854115

[20] Herrera D Sanz M Kebschull M Jepsen S Sculean A Berglundh T Treatment of stage iv periodontitis: the efp S3 level clinical practice guideline. J Clin Periodontol. (2022) 49(Suppl 24):4–71. 10.1111/jcpe.13639 35688447

[21] von Elm E Altman DG Egger M Pocock SJ Gøtzsche PC Vandenbroucke JP. The strengthening the reporting of observational studies in epidemiology (strobe) statement: guidelines for reporting observational studies. J Clin Epidemiol. (2008) 61(4):344–9. 10.1016/j.jclinepi.2007.11.008 18313558

[22] Werner N Heck K Walter ED Ern C Bumm CV Folwaczny M. Probing pocket depth reduction after nonsurgical periodontal therapy: tooth-related factors. J Periodontol. (2023) 00:1–11. 10.1002/JPER.23-0285 37436696

[23] Papapanou PN Sanz M Buduneli N Dietrich T Feres M Fine DH Periodontitis: consensus report of workgroup 2 of the 2017 world workshop on the classification of periodontal and peri-implant diseases and conditions. J Clin Periodontol. (2018) 45(S20):S162–S70. 10.1111/jcpe.12946 29926490

[24] Heym R Krause S Hennessen T Pitchika V Ern C Hickel R. A new model for training in periodontal examinations using manikins. J Dent Educ. (2016) 80(12):1422–9. 10.1002/j.0022-0337.2016.80.12.tb06229.x 27934667

[25] Van der Weijden GA Timmerman MF Saxton CA Russell JI Huntington E Van der Velden U Intra-/inter-examiner reproducibility study of gingival bleeding. J Periodontal Res. (1994) 29(4):236–41. 10.1111/j.1600-0765.1994.tb01217.x 7932016

[26] Miller SC. Textbook of Periodontia (Oral Medicine). Philadelphia: Blakiston (1950). p. 900.

[27] Schiffman E Ohrbach R Truelove E Look J Anderson G Goulet JP Diagnostic criteria for temporomandibular disorders (dc/tmd) for clinical and research applications: recommendations of the international rdc/tmd consortium network* and orofacial pain special interest group†. J Oral Facial Pain Headache. (2014) 28(1):6–27. 10.11607/jop.1151 24482784 PMC4478082

[28] Bumm CV Ern C Folwaczny J Wölfle UC Heck K Werner N Periodontal grading—estimation of responsiveness to therapy and progression of disease. Clin Oral Investig. (2024) 28(5):289. 10.1007/s00784-024-05678-3 PMC11062956 38691197

[29] Nibali L Pometti D Tu Y-K Donos N. Clinical and radiographic outcomes following non-surgical therapy of periodontal infrabony defects: a retrospective study. J Clin Periodontol. (2011) 38(1):50–7. 10.1111/j.1600-051X.2010.01648.x 21091528

[30] Tsitoura E Tucker R Suvan J Laurell L Cortellini P Tonetti M. Baseline radiographic defect angle of the intrabony defect as a prognostic indicator in regenerative periodontal surgery with enamel matrix derivative. J Clin Periodontol (2004) 31(8):643–7. 10.1111/j.1600-051X.2004.00555.x 15257742

[31] Krois J Ekert T Meinhold L Golla T Kharbot B Wittemeier A Deep learning for the radiographic detection of periodontal bone loss. Sci Rep. (2019) 9(1):8495. 10.1038/s41598-019-44839-3 31186466 PMC6560098

[32] Gonzalez YM Schiffman E Gordon SM Seago B Truelove EL Slade G Development of a brief and effective temporomandibular disorder pain screening questionnaire: reliability and validity. J Am Dent Assoc. (2011) 142(10):1183–91. 10.14219/jada.archive.2011.0088 21965492 PMC4527600

[33] Lövgren A Parvaneh H Lobbezoo F Häggman-Henrikson B Wänman A Visscher CM. Diagnostic accuracy of three screening questions (3q/tmd) in relation to the dc/tmd in a specialized orofacial pain clinic. Acta Odontol Scand. (2018) 76(6):380–6. 10.1080/00016357.2018.1439528 29448865

[34] Lövgren A Visscher CM Häggman-Henrikson B Lobbezoo F Marklund S Wänman A. Validity of three screening questions (3q/tmd) in relation to the dc/tmd. J Oral Rehabil. (2016) 43(10):729–36. 10.1111/joor.12428 27573533

[35] Jeon HM Ahn YW Jeong SH Ok SM Choi J Lee JY Pattern analysis of patients with temporomandibular disorders resulting from unilateral mastication due to chronic periodontitis. J Periodontal Implant Sci. (2017) 47(4):211–8. 10.5051/jpis.2017.47.4.211 28861285 PMC5577439

[36] Kirveskari P Alanen P. Association between tooth loss and tmj dysfunction. J Oral Rehabil. (1985) 12(3):189–94. 10.1111/j.1365-2842.1985.tb00635.x 3859625

[37] Needleman I Nibali L Di Iorio A. Professional mechanical plaque removal for prevention of periodontal diseases in adults – systematic review update. J Clin Periodontol (2015) 42(S16):S12–35. 10.1111/jcpe.12341 25495962

[38] Axelsson P Lindhe J. Effect of controlled oral hygiene procedures on caries and periodontal disease in adults. Results after 6 years. J Clin Periodontol. (1981) 8(3):239–48. 10.1111/j.1600-051x.1981.tb02035.x 6947990

[39] Humphrey SP Lindroth JE Carlson CR. Routine dental care in patients with temporomandibular disorders. J Orofac Pain. (2002) 16(2):129–34. 12043519

[40] Beddis HP Davies SJ Budenberg A Horner K Pemberton MN. Temporomandibular disorders, trismus and malignancy: development of a checklist to improve patient safety. Br Dent J. (2014) 217(7):351–5. 10.1038/sj.bdj.2014.862 25303582

[41] Macapanpan LC Weinmann JP. The influence of injury to the periodontal membrane on the spread of gingival inflammation. J Dent Res. (1954) 33(2):263–72. 10.1177/00220345540330021301 13152264

[42] Jiao J Shi D Cao Z-q Meng H-x Lu R-f Zhang L Effectiveness of non-surgical periodontal therapy in a large Chinese population with chronic periodontitis. J Clin Periodontol. (2017) 44(1):42–50. 10.1111/jcpe.12637 27726174

[43] Bertl K Savvidis P Kukla EB Schneider S Zauza K Bruckmann C Including dental professionals in the multidisciplinary treatment team of head and neck cancer patients improves long-term oral health Status. Clin Oral Investig. (2022) 26(3):2937–48. 10.1007/s00784-021-04276-x PMC8600104 34792667

